# 功能化磁性纳米材料在糖蛋白及糖肽富集中的研究进展

**DOI:** 10.3724/SP.J.1123.2021.08012

**Published:** 2021-09-08

**Authors:** Wenjie GAO, Yu BAI, Huwei LIU

**Affiliations:** 北京大学化学与分子工程学院, 北京分子科学国家实验室, 北京 100871; Beijing National Laboratory of Molecular Science, College of Chemistry and Molecular Engineering, Peking University, Beijing 100871, China; 北京大学化学与分子工程学院, 北京分子科学国家实验室, 北京 100871; Beijing National Laboratory of Molecular Science, College of Chemistry and Molecular Engineering, Peking University, Beijing 100871, China; 北京大学化学与分子工程学院, 北京分子科学国家实验室, 北京 100871; Beijing National Laboratory of Molecular Science, College of Chemistry and Molecular Engineering, Peking University, Beijing 100871, China

**Keywords:** 功能化磁性纳米材料, 富集, 糖蛋白, 糖肽, 综述, functionalized magnetic nanomaterials, enrichment, glycoproteins, glycopeptides, review

## Abstract

蛋白质糖基化作为最重要的翻译后修饰之一,在生物体诸如细胞信号转导、蛋白质翻译调控、免疫应答等诸多生命过程中发挥重要作用。此外,蛋白质的异常糖基化还与肿瘤等疾病的发生发展密切相关,这为以糖蛋白为目标的疾病生物标志物的发现提供了可能。尽管质谱已经成为糖蛋白质组学的重要分析工具,但糖肽的低丰度和低电离效率使得其直接质谱分析仍面临挑战。在糖蛋白质组学研究中,从复杂的生物样品中富集糖蛋白和糖肽是重要的环节。磁性固相萃取(MSPE)是一种操作简单、成本低和萃取效率高的样品预处理方法。在磁性固相萃取中,磁性吸附剂是影响萃取效果的关键,将功能化磁性纳米材料作为吸附剂进行糖蛋白质组学研究已经得到广泛应用。该文综述了糖分子、离子液体、凝集素、硼酸亲和配体、金属有机框架、共价有机骨架等功能化磁性纳米材料的制备及其在糖蛋白及糖肽富集中的应用。上述功能化磁性纳米材料具有高比表面积、大量作用位点等特点,其富集机理包括亲水相互作用色谱、凝集素亲和作用色谱、硼酸化学法和肼化学法等,主要应用于血清、血浆、细胞、组织、唾液等样品的糖蛋白和糖肽的富集。该文引用了近十年来发表的约90篇源于科学引文索引(SCI)与中文核心期刊的相关论文,并于文末对磁性纳米材料在糖蛋白和糖肽富集领域的发展趋势进行了展望。

## Abstract (en)

Protein glycosylation is among the most common and important post-translational modifications, and plays an important regulatory role in many biological processes, including signal transduction, protein translation, and immune response. Abnormal protein glycosylation is also associated with numerous diseases, suggesting that glycoproteins may offer an array of useful disease biomarkers. Mass spectrometry (MS) has become an important analytical tool in glycoproteomics. However, the low abundance and weak ionization efficiency of glycopeptides have hindered direct mass spectrometric analyses, which remain considerably challenging. Glycoprotein and glycopeptide enrichment from complex biological samples is an important step in glycoproteomics. Diverse methods have recently been developed for specific glycoprotein and glycopeptide enrichment, including hydrophilic interaction liquid chromatography (HILIC), lectin affinity chromatography, boronate affinity chromatography, and hydrazide functional affinity chromatography. A variety of enrichment materials designed for the above strategies have been developed to meet the requirement of enriching low abundance glycoproteins and glycopeptides in complex samples. Magnetic solid phase extraction (MSPE) is an efficient sample pretreatment technology that offers advantages of simple operation, low cost, and high extraction efficiency. Functionalized magnetic nanomaterials have been widely used as adsorbents in glycoproteome studies. Since magnetic adsorbent is a key factor in MSPE, in this review, the preparation of magnetic nanomaterials functionalized with sugars, ionic liquids, lectins, boronate affinity ligands, metal organic frameworks, and covalent organic frameworks, and their applications in glycoprotein and glycopeptide enrichment are summarized. These functional magnetic nanomaterials possess high specific surface area and a large number of active adsorption sites, allowing different enrichment mechanisms, including HILIC, lectin affinity chromatography, and boronate and hydrazide functional affinity chromatography. These functional magnetic nanomaterials are mainly used to enrich glycoproteins and glycopeptides in serum, plasma, cells, tissues, saliva and other biological samples. Nearly 90 papers published in the last decade from the Science Citation Index (SCI) and Chinese core journals have been cited in this paper. Finally, the development and prospects of magnetic nanomaterials in glycoprotein and glycopeptide enrichment are also discussed.

作为生物体最重要、最常见的蛋白质翻译后修饰之一,蛋白质糖基化在细胞间相互作用、信号转导、免疫应答等多种生物过程中发挥了重要作用。异常的蛋白质糖基化与许多疾病有关,例如神经退行性疾病和癌症等^[1,2,3]^。截至目前,美国食品药品监督管理局(FDA)批准的肿瘤标志物大部分是糖蛋白,例如糖抗原199(CA19-9)、前列腺特异性抗原(PSA)、甲胎蛋白(AFP)等^[4,5,6]^。此外,许多糖基化的膜蛋白、细胞外蛋白等可以用作药物治疗的靶点,例如表皮生长因子受体-2(Her 2)可作为乳腺癌治疗的靶点^[7]^。因此,蛋白质糖基化已经成为一个跨化学、生物学和临床医学等诸多学科的交叉研究领域。

通常,糖蛋白质组学分析对象主要包含糖蛋白和糖肽。在糖蛋白水平,完整糖蛋白需首先通过不同的分离手段实现富集,然后进行后续的分析和检测。未知糖蛋白需经过多肽序列测定、糖基化位点的鉴定、聚糖组成和结构表征等。糖肽分析常见的策略是鸟枪法,即将糖蛋白酶切后对糖肽进行富集,进而利用质谱检测,该法已被广泛用于基于糖蛋白组学的生物标志物的发现研究。然而,蛋白质的糖基化修饰异常复杂,即使同一个糖基化位点,也可能存在不同的糖链修饰,即糖链的微观不均一性,这一现象的存在使得基于质谱法分析复杂样品中的糖肽仍然面临挑战^[8]^。此外,在复杂生物样品中,糖蛋白多为低丰度蛋白,且酶切后的糖肽只占所有酶解多肽的约2%~5%^[9]^。因此,在质谱分析之前对样品中的糖蛋白和糖肽进行富集分离十分必要。

近年来,研究人员已经提出了多种针对糖蛋白和糖肽富集的方法,包括亲水相互作用色谱法、凝集素亲和色谱法、硼酸亲和色谱法、酰肼化学法等^[10,11,12,13]^。将磁性纳米材料作为磁性吸附剂用于样品前处理的技术称为磁性固相萃取(magnetic solid phase extraction, MSPE),已经成为一种广泛使用的样品前处理技术。相比于其他的样品前处理技术,如固相萃取、液液萃取等,MSPE具有操作简便、有机试剂使用少、萃取效率高等优点。在磁性固相萃取技术中,磁性纳米吸附剂是影响富集和选择性的关键。通过在磁性纳米材料表面进行修饰或者将其与其他材料进行结合,可以提高材料的物理和化学稳定性。近年来,功能化的磁性纳米复合材料在糖蛋白和糖肽的富集领域应用较为广泛。

本文对近十年来基于各种功能化的磁性纳米材料的糖蛋白和糖肽富集的相关研究工作进行综述,便于相关人员更好地了解该方面的研究进展。此外,我们还对磁性纳米材料在糖蛋白和糖肽富集中的前景进行了展望。

## 1 基于亲水相互作用的磁性纳米材料

糖蛋白和糖肽上的糖链中存在大量羟基,使得糖蛋白和糖肽相比于非糖蛋白和非糖肽具有更强的亲水性,这为亲水相互作用色谱法用于糖蛋白和糖肽富集奠定了基础。亲水相互作用色谱的富集机理为待测分子在亲水固定相形成的富水层与高有机流动相之间发生分配,进而实现分离。此外,氢键和静电相互作用在亲水相互作用色谱中也发挥了作用。在基于亲水相互作用的磁性纳米材料用于糖蛋白和糖肽富集的方法中,常用作功能化的亲水介质主要包含不同类型的糖分子、亲水多孔有机框架和两性离子等。

### 1.1 糖分子功能化的磁性纳米材料

糖分子之间的相互作用在生物体中十分常见,例如细胞膜表面配体和受体糖蛋白的相互作用。受到这一启发,不同糖分子功能化的磁性纳米材料,例如,葡萄糖、羧甲基-*β*-环糊精、麦芽糖、壳聚糖等,已经被广泛用于糖蛋白和糖肽的富集^[14,15,16,17,18]^。Li等^[19]^利用磁纳米颗粒表面的Fe^3+^/Fe^2+^可以与磷酸基团螯合的原理,将磷酸葡萄糖修饰到介孔硅包被的磁纳米颗粒上。利用该材料良好的亲水性和尺寸排阻能力,从健康志愿者和胃癌病人唾液中分别富集到39条和25条内源性糖肽,在糖肽组学生物标志物发现方面显示了巨大的潜力。

据报道,在磁性纳米材料上接枝更多的亲水官能团可以使其在应对高度复杂的生物样品时有更好的表现^[20]^。为了增加亲水分子的修饰密度,提高材料对糖肽的吸附容量,研究人员常使用树枝状分子或聚合分子来增加亲水分子的结合位点。Bi等^[21]^在磁纳米颗粒表面缀合了高亲水和多功能基团的聚乙烯亚胺(PEI)分子,然后再结合麦芽糖官能团,制备了超亲水的磁性聚合物刷,图1为该材料合成及富集示意图。该材料具有较大的吸附容量(200 mg/g),亲水聚合物刷的存在降低了材料对非糖肽的吸附。Zhan等^[22]^使用PEI作为接枝分子,通过静电自组装将透明质酸固定到磁性氧化石墨烯上。该材料的吸附容量达到了300 mg/g,选择性也达到了免疫球蛋白G(IgG):牛血清白蛋白(BSA)=1:1000(物质的量之比)的水平。

**图1:**
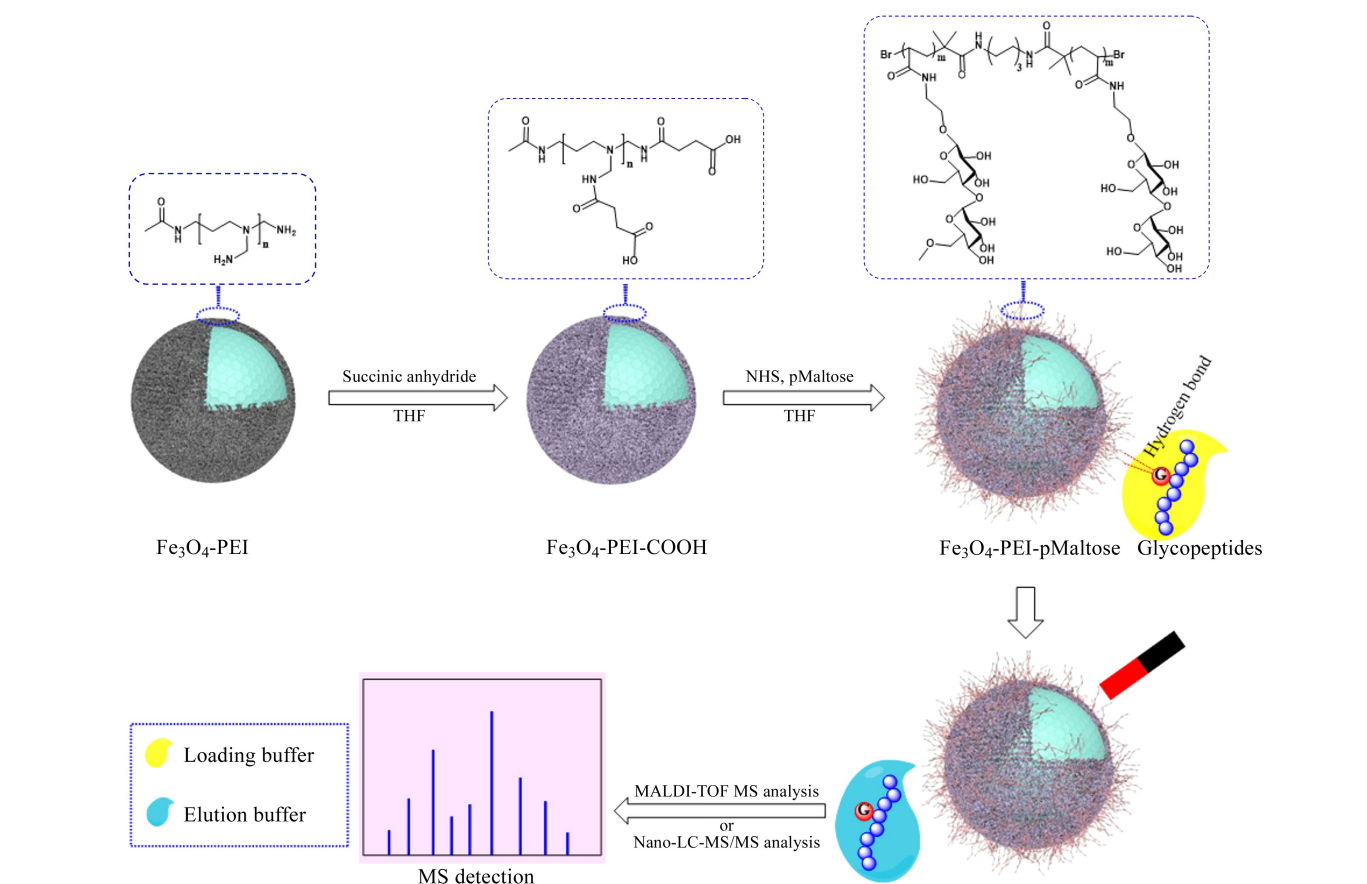
Fe_3_O_4_-PEI-pMaltose纳米颗粒的制备及*N*-连接糖肽选择性富集的示意图^[21]^

### 1.2 亲水多孔有机框架材料功能化的磁性纳米材料

多孔有机框架材料,例如金属有机框架材料(MOFs)和共价有机框架材料(COFs),由于其高孔隙率、大比表面积和结构多样性的特点,近来引起了研究者的广泛关注。在样品前处理应用中,已经有大量研究利用亲水多孔有机框架材料对磁性纳米材料进行功能化,进而用于糖蛋白和糖肽的富集等^[23,24,25,26,27,28,29]^。

有机框架材料常通过外延生长或自组装的方法修饰到磁性纳米材料上,因此形成的多是核壳结构的复合材料。Zheng等^[30]^通过层层自组装的方法制备了羰基功能化的磁性锆金属有机框架材料。基于该材料的亲水性和锆的金属亲和色谱,作者将其用于不同尺寸外泌体中糖蛋白质组和磷酸蛋白质组的分析。实验结果显示,不同大小外泌体中包裹的蛋白质的糖基化和磷酸化水平有显著差异。

Wu等^[31]^合成了含3个羧基的亲水单体并将其用于制备磁性亲水共价有机框架材料。该材料因其优异的亲水性、大量的纳米孔结构和快速的磁响应,成功用于唾液中内源性糖肽的富集。除了糖基化修饰,磷酸化修饰也是一种重要的蛋白质翻译后修饰,因此能够同时实现两种翻译后修饰多肽的富集成为一些研究者的目标。Zheng等^[32]^制备了一种双功能镓离子固定磁性柱[5]芳烃超分子有机框架材料,用于同时富集糖肽和磷酸化多肽。该材料表面丰富的磺酸基使其具有强的亲水性,可以用于糖肽的富集;而磷酸化多肽可以通过与Ga^3+^之间的螯合作用实现分离富集。利用该材料可从小鼠肝脏中富集到1006条糖肽(665个糖蛋白)和1332条磷酸化多肽(908个磷酸化蛋白)。

### 1.3 两性离子功能化的磁性纳米材料

两性离子是一类带有正电和负电基团、整体维持电中性的分子。两性离子具有强的水化能力,通过静电诱导水合作用,在两性离子表面形成一层致密的水层。由于优良的亲水性和生物相容性,两性离子功能化磁性纳米材料常被用于糖肽的富集^[33,34,35,36]^。Ji等^[37]^使用简单的一步蒸馏沉淀法制备了两性离子[2-(甲基丙烯酰基氧基)乙基]二甲基-(3-磺酸丙基)氢氧化铵聚合物(PMSA)包被的磁性纳米颗粒(Fe_3_O_4_@PMSA),并将其用于富集血清中的糖肽,可从1 μL血清中富集到158个糖蛋白中419条糖肽。

半胱氨酸(Cys)和谷胱甘肽(GSH)作为常见的两性离子,被广泛地用于修饰磁性纳米材料,修饰后的材料进而用于糖肽的富集^[38,39,40,41]^。Feng等^[42]^基于金属有机配位原理通过一步法将半胱氨酸修饰到磁性纳米颗粒表面。通过该简便方法制备的磁性纳米材料在富集标准糖蛋白的酶解糖肽时显示了优异的灵敏度(25 amol/μL)和稳定性(室温下放置1个月)。然而,该材料的吸附容量不甚理想,这主要归因于功能化修饰的官能团密度不够高。有研究者利用这两类分子都含有巯基的特点,通过金硫键将其固定到金纳米颗粒上,进一步提高功能基团的密度^[43,44]^。Liu等^[45]^基于金硫键将GSH枝接到金纳米颗粒固定的磁性MOFs表面,制备了mMOF@Au@GSH。得益于GSH的亲水性和UiO-66-NH_2_的大比表面积,该材料获得了较大的吸附容量(140 mg/g)。

有研究证实内源性糖肽在多种病理和生理过程中发挥重要作用。复杂生物基质中内源性糖肽丰度低,干扰物多,这增加了内源性糖肽分析的难度。Wang等^[46]^制备了亲水的磁性介孔硅纳米材料用于富集人唾液中的内源性糖肽。介孔硅的存在可以通过尺寸效应排除掉大的蛋白质的干扰,从人唾液中共富集到40个内源性糖肽。Luo等^[47]^通过合成后修饰的方法制备了GSH功能化的磁性共价有机框架材料,将其用于人唾液中内源性糖肽的富集,可从10 μL人唾液中富集到143个内源性糖肽。

### 1.4 其他

除了常用的糖分子、亲水有机框架和两性离子,离子液体和一些带羟基、羧基等的分子(例如柠檬酸、琥珀酸、植酸、聚乙二醇等)也被用于制备磁性纳米材料,进而应用于糖蛋白和糖肽的富集^[48,49,50,51,52,53,54,55]^。Sun等^[56]^设计并合成了亚氨基乙二酸功能化的磁性介孔硅纳米材料。由于大量羧基的存在和介孔材料的大比表面积,该材料具有优异的亲水性。利用人血清作为复杂实际样品,可从2 μL血清中富集到140个糖蛋白中的424条糖肽。该课题组还利用巯基和钛之间的相互作用,合成了巯基琥珀酸修饰的磁性介孔二氧化钛纳米材料^[57]^,成功用于糖肽和磷酸化多肽的同时富集。

除了将亲水材料包被在磁性纳米材料表面来制备功能化磁性纳米材料以外,还可以通过将磁性纳米材料生长在具有大比表面积的基底材料上,再进一步功能化修饰。Wang等^[58]^将亲水MOFs生长到磁性石墨烯复合物上,制备了厚度可控的亲水磁性纳米材料。制备的复合物具有良好的检出限(0.1 fmol/μL)和尺寸排阻性能(辣根过氧化物酶(HRP)酶解液:BSA:HRP=1:500:500,质量比),以及较大的吸附容量(150 mg/g)和高的回收率(大于90%)。该材料从膀胱癌患者尿液中富集到406条糖肽,对应于185个糖蛋白。

## 2 基于凝集素亲和色谱的磁性纳米材料

凝集素能够识别某一特殊结构的单糖或聚糖中特定的序列,因此常被用作糖蛋白和糖肽富集的亲和探针^[59,60]^。2010年,Hill课题组^[61]^开发了凝集素磁珠阵列(LeMBA)技术用于糖蛋白生物标志物的发现研究。该技术主要工作流程是使用不同凝集素固定的磁珠用于血清中糖蛋白的富集,对富集到的蛋白进行胰酶酶切后,用液相色谱-质谱技术对富集的糖蛋白进行鉴定。此后,该课题组对这一技术进行了改进和完善,并将其用于人血清和唾液中糖蛋白的富集和鉴定^[62,63]^。作为应用,研究者将该技术用于食管腺癌糖蛋白生物标志物的研究,并发现了载脂蛋白B-100、补体成分C9和凝溶胶蛋白这3种蛋白质为可能的候选标志物^[64]^。

由于凝集素对于糖结构的识别比较单一,没有普适性,为了尽可能多的富集糖蛋白,常需要使用多种凝集素联用的方法。图2所示为Waniwan等^[65]^使用伴刀豆蛋白A(ConA)、金橙黄网胞盘菌凝集素(AAL)和接骨木凝集素(SNA)3种凝集素包被的磁珠,用于富集非小细胞肺癌细胞株PC-9和其耐药细胞株PC9-IR膜蛋白中的糖肽,分别从两种细胞株中富集到2290和2767条特异性糖肽,且PC9-IR细胞株中的糖肽多是岩藻糖化和唾液酸化的糖肽。

**图2:**
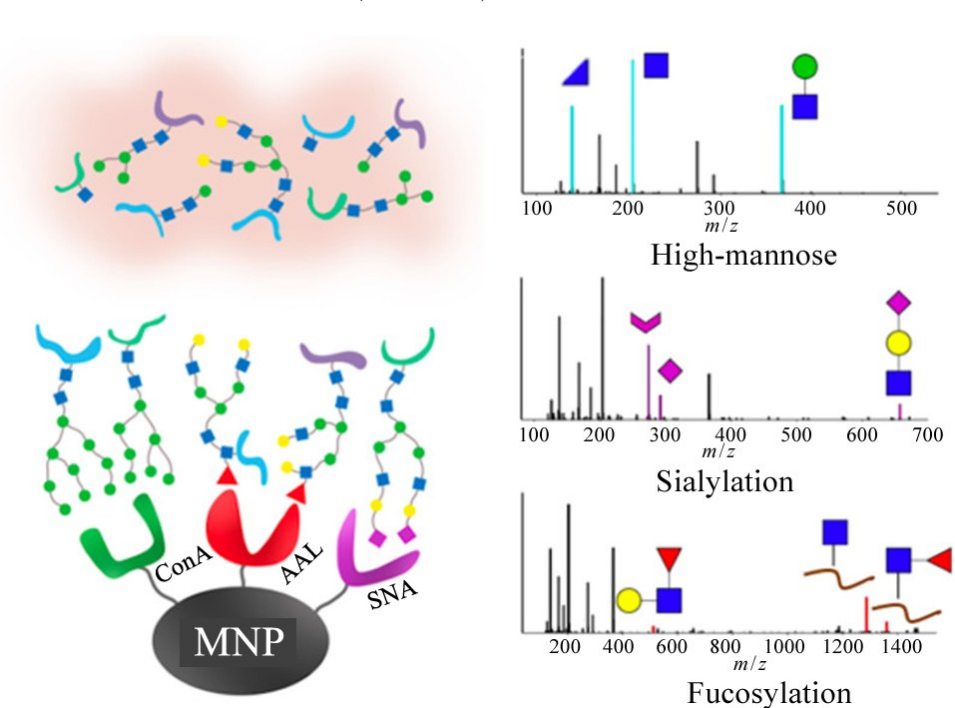
3种凝集素包被磁珠用于糖肽富集^[65]^

## 3 基于硼酸化学的磁性纳米材料

硼酸化学法富集糖蛋白和糖肽的原理如下:在碱性条件下,硼酸与糖链的顺式二醇反应形成五元或六元环酯,并且在酸性条件下该反应可发生逆向移动,实现环酯断裂,进而释放富集的糖蛋白或糖肽^[66]^。

含有多种官能团的硼酸分子被用于磁性材料的功能化,例如4-巯基苯硼酸、氨基苯硼酸、3-醛基苯硼酸等^[67,68,69,70,71,72]^。Yao等^[73]^利用Fe和巯基间的相互作用,通过简单的一步法反应在磁性纳米颗粒表面修饰了4-巯基苯硼酸。该材料可从1 μL血清中富集到来源于93个糖蛋白的230条肽段。Sun等^[74]^制备了硼酸修饰的介孔二氧化钛(TiO_2_)包被的磁性纳米颗粒。介孔TiO_2_的花式结构提供了更多的修饰位点,与光滑的二氧化硅材料包被的磁性纳米材料相比,其在吸附容量上有了明显提升。此外,4价钛离子的强吸电子效应,使得硼酸在6.0~9.0的宽pH范围内对糖蛋白有较好的亲和作用。

由于硼酸与顺式二醇之间是弱亲和作用,对于低丰度糖蛋白及糖肽的富集仍具有挑战性。为了解决这一问题,Liu等^[75]^率先提出了多价协同结合的策略,并利用该策略合成了聚酰胺-胺型树枝状大分子(PAMAM)硼酸功能化的磁性纳米颗粒。该材料对于糖蛋白的解离常数达到了10^-5^ ~10^-6^mol/L,比单硼酸亲和材料的结合力提高了3~4个数量级。随着结合能力的显著提高,该材料不仅能够有效富集低丰度糖蛋白,材料富集容量和糖蛋白富集速度也得到了提升。基于该策略,研究者开发并制备了多种多样的硼酸功能化磁纳米材料用于糖蛋白和糖肽的富集^[76,77,78]^。Li等^[69]^使用分支聚乙烯亚胺辅助制备了高硼酸密度的磁性纳米颗粒,该材料对于糖肽的解离常数达到了10^-6^~10^-7^mol/L。由于其与糖蛋白的高结合能力,该材料可以富集浓度低至2×10^-15^mol/L的痕量蛋白质。Xiao等^[79]^基于多价协同富集策略,通过优化硼酸功能化分子,制备了高硼酸密度的功能化磁性纳米材料(见图3)。

**图3:**
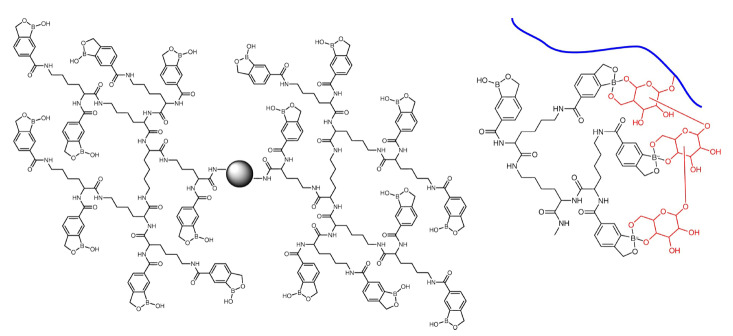
树状分子缀合硼酸衍生物(DBA)磁珠与糖肽协同相互作用的原理图^[79]^

该材料可用于酵母、小鼠脑组织、细胞等样品的大规模糖蛋白质组分析。值得一提的是,得益于多价协同作用,该材料还可用于完整*O*-糖肽的富集及鉴定。

硼酸富集法在富集糖蛋白时需要在碱性条件下完成与蛋白质的结合,这可能会导致脆弱糖蛋白的降解并影响富集效果,因此合成低p*K*_a_值的配体对实现糖蛋白质组学深度分析十分必要^[80,81]^。Zhang等^[82]^使用低p*K*_a_的苯并氧杂硼戊环(benzoboroxole)作为配体制备了功能化的磁性核壳微球。这一纳米复合材料具有非特异性吸附低、富集时间短(10 min)、磁响应性高的特点,并可以实现生理条件下(pH 7.4)牛血清中的糖蛋白的富集。Wu等^[83]^制备了3-羧基苯并氧杂硼戊环(CBX)功能化的聚乙烯亚胺修饰的磁性氧化石墨烯纳米复合材料。由于CBX的p*K*_a_约为6.9,该复合材料可以在生理条件下富集糖蛋白。该材料成功应用于人血浆糖蛋白质组分析,富集到碱性敏感的凝血酶蛋白(F2),该蛋白质未在以前的硼酸功能化材料富集中检测到。

## 4 基于酰肼化学的磁性纳米材料

与硼酸亲和色谱法类似,酰肼化学法富集糖蛋白和糖肽也是利用糖链上的多羟基结构,与之不同的是,需将糖链上的顺式二醇氧化为醛基,醛基进一步与固定相上的酰肼反应形成腙,从而被富集。洗掉未反应的非糖肽或非糖蛋白后,利用*N*-糖苷酶F(PNGase F)等糖苷酶将糖肽从固定相上酶切并释放。Zhang等^[12]^首次使用该方法富集人血清中糖蛋白。后来,该课题组又合成了肼功能化的磁珠,并使用微孔板结合自动处理系统实现了高通量临床样本的筛选^[84]^。

目前,肼功能化磁珠已经商品化,并被应用于糖肽定量分析、黑曲霉分泌组和完整细胞的糖基化位点分析、卵巢癌糖蛋白生物标志物等研究^[85,86,87,88]^。然而,肼功能化材料存在修饰密度低的问题。为了提高材料表面肼功能基团的密度,Liu等^[89]^合成了一种聚甲基丙烯肼(PMAH)功能化的磁性纳米复合材料(Fe_3_O_4_@PMAH),图4为其合成过程。与商品化的肼功能化树脂相比较,该肼功能化磁性材料在富集标准糖蛋白时获得了5倍的质谱信噪比提升。将该材料用于结肠癌病人血清中糖肽的富集,共鉴定到来自63个糖蛋白的175条特异性糖肽。

**图4:**
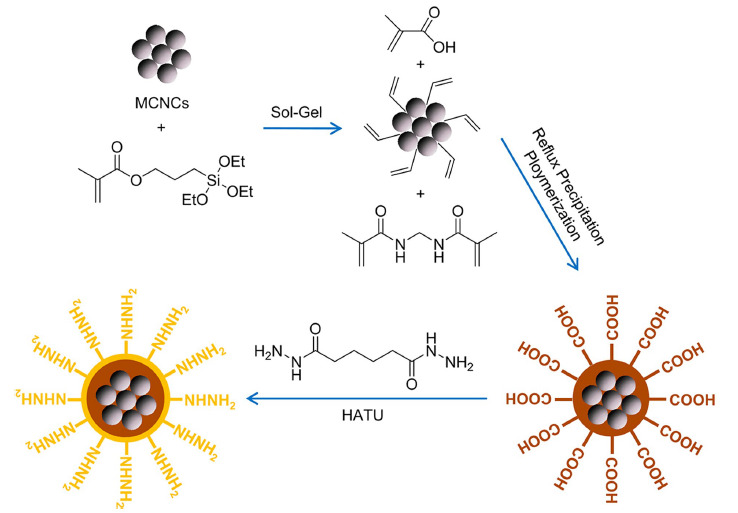
Fe_3_O_4_@PMAH核壳磁性纳米复合材料的合成过程^[89]^

肼化学法富集糖蛋白及糖肽具有专一性强和无位点偏向性的优点,但也存在着诸多问题,比如需要经多步化学反应,增加了反应时间和样品的复杂程度;肼对糖链的水解和脱乙酰作用会造成糖链结构的破坏,导致该方法适合糖基化位点的分析,而丢失糖链结构信息。

## 5 结论与展望

作为一种重要的翻译后修饰,蛋白质糖基化已经得到了科研人员的广泛关注。由于糖蛋白及糖肽在复杂生物样品及临床样本中的低丰度,样品分析前进行富集成为必不可少的步骤。功能化修饰磁性纳米材料具有简便、快速和高效的特点,广泛应用于糖蛋白和糖肽富集,该方法有力推动了糖蛋白质组学的发展。虽然通过生物信息学估计,有超过50%的哺乳动物细胞蛋白是糖蛋白,但是现在数据库中注释为糖蛋白的仅有约10%,这表明蛋白质糖基化的研究仍然任重道远。

对于糖蛋白质组学分析,选择性、回收率、覆盖度、吸附容量和重现性是衡量富集材料的重要参数。而对于大规模的生物和临床样本来说,高通量分析方法的建立迫在眉睫。随着材料科学的发展,新型亲水材料的设计合成将为糖蛋白的鉴定和深度分析提供新的解决方案和方法。此外,新的富集策略和分析鉴定技术的进步和完善,将在基于糖蛋白质组学疾病生物标志物的发现及药物靶点的研究等方面发挥重要作用。
